# The Role of Tumor Necrosis Factor Associated Factors (TRAFs) in Vascular Inflammation and Atherosclerosis

**DOI:** 10.3389/fcvm.2022.826630

**Published:** 2022-02-17

**Authors:** Mark Colin Gissler, Peter Stachon, Dennis Wolf, Timoteo Marchini

**Affiliations:** ^1^Cardiology and Angiology, Medical Center, University of Freiburg, Freiburg im Breisgau, Germany; ^2^Faculty of Medicine, University of Freiburg, Freiburg im Breisgau, Germany; ^3^Universidad de Buenos Aires, CONICET, Instituto de Bioquímica y Medicina Molecular (IBIMOL), Facultad de Farmacia y Bioquímica, Buenos Aires, Argentina

**Keywords:** TRAF, atherosclerosis, immunity, inflammation, TNF, signaling

## Abstract

TNF receptor associated factors (TRAFs) represent a family of cytoplasmic signaling adaptor proteins that regulate, bundle, and transduce inflammatory signals downstream of TNF- (TNF-Rs), interleukin (IL)-1-, Toll-like- (TLRs), and IL-17 receptors. TRAFs play a pivotal role in regulating cell survival and immune cell function and are fundamental regulators of acute and chronic inflammation. Lately, the inhibition of inflammation by anti-cytokine therapy has emerged as novel treatment strategy in patients with atherosclerosis. Likewise, growing evidence from preclinical experiments proposes TRAFs as potent modulators of inflammation in atherosclerosis and vascular inflammation. Yet, TRAFs show a highly complex interplay between different TRAF-family members with partially opposing and overlapping functions that are determined by the level of cellular expression, concomitant signaling events, and the context of the disease. Therefore, inhibition of specific TRAFs may be beneficial in one condition and harmful in others. Here, we carefully discuss the cellular expression and signaling events of TRAFs and evaluate their role in vascular inflammation and atherosclerosis. We also highlight metabolic effects of TRAFs and discuss the development of TRAF-based therapeutics in the future.

## Atherosclerosis is a Chronic Inflammatory Disease of Arteries

Atherosclerosis is a disease of medium- to large-sized arteries that leads to the build-up of vessel occluding atherosclerotic plaques. Endothelial erosion and plaque rupture represent frequent and fatal complications of atherosclerosis that may lead to the formation of arterial thrombi, vessel occlusion, and subsequent tissue ischemia (1, 2). The prevalence of diseases caused by atherosclerosis, including coronary heart disease, stroke, and peripheral arterial occlusive disease, continues to rise and doubled from 271 million in 1990 to 523 million in 2019 (3, 4). The accumulation of low-density lipoprotein (LDL) cholesterol in the subintimal space of arteries triggered by cardiovascular risk factors, such as hypertension and diabetes mellitus, is the major culprit of atherosclerotic disease. Beyond the passive accumulation of LDL in the atherosclerotic plaque and its uptake by tissue-resident macrophages that fuels plaque growth, cumulating evidence of numerous preclinical and clinical studies have stablished that atherosclerosis is a chronic inflammatory and immune-driven disease of the arterial wall (5). This response involves stromal cells, such as endothelial cells (EC) and smooth muscle cells (SMC), and cells of the innate and adaptive immune system. Recent evidence suggests that a part of the immune response in atherosclerosis is caused by autoimmunity against LDL and Apolipoprotein B (6, 7). Immune cells accumulate in the subintimal space of atherosclerotic arteries and interact with stromal cells by direct cellular contact and cytokines (8, 9). It is now established that inflammation is a local and systemic process that promotes atherosclerosis at all stages, from initial endothelial dysfunction to thrombotic complications of acute plaque rupture (10). Therapeutic inhibition of inflammation has the potential to prevent atherosclerosis progression and improve cardiovascular outcomes, as recently demonstrated by a neutralizing antibody against the pro-inflammatory master cytokine interleukin (IL)-1β by canakinumab (11), the pro-inflammatory cytokine IL-6 (12), and by colchicine that has broad anti-inflammatory properties (13, 14). Neutralization of other pro-inflammatory cytokines, such as of TNF-α and IL-17, revealed inconsistent effects on cardiovascular end-points (15) or worsened cardiovascular risk factors as exemplified by the increased risk for hypertension during anti-TNF therapy of rheumatoid arthritis (16). In addition, potential side-effects of live-long immune-modulating therapy, such as infection, have not yet been systematically evaluated albeit first safety signals from canakinumab and colchicine trials were promising (17, 18). It is therefore instrumental to define alternative therapeutic targets that are confined to atherosclerosis-relevant cell types and broadly regulate inflammatory signaling cascades without the risk of severe immunosuppression. Mostly preclinical evidence has suggested that TRAFs may represent such inflammatory targets.

## Pro-Inflammatory and Cellular Networks in Atherosclerotic Disease

It is now well-established that inflammatory signaling cascades are potent modulators of atherosclerosis (19). Besides their clinical use as biomarkers (20), several cytokines have been shown to orchestrate the inflammatory response in the atherosclerotic plaque, such as TNF-α, IL-1β IL-6, and IL-12 (21). Cytokines are soluble factors that are locally produced in atherosclerotic plaques and in remote organs, mostly, yet not exclusively, by immune cells (22). They circulate through the blood stream and modulate immune mechanisms in a plethora of stromal and immune cells in the plaque and cardiometabolic key organs. Distinct outcomes of cytokine signaling pathways are determined by their specific binding to a range of receptors with cell type-specific expression, and partially synergistic and antagonistic functions. Cytokines are involved in all stages of atherosclerotic disease, from initial endothelial dysfunction to pro-thrombotic events (21). Consistently, it has been established that a therapeutic modulation of some pro-inflammatory master cytokines and their receptors may efficiently interfere with atherosclerosis development in preclinical mouse models. CD40L, TNF-α, and IL-1β have been extensively evaluated in experimental atherosclerosis (23–25) and the development of underlying cardiometabolic risk factors (26, 27). One relevant limitation of targeting single receptor-ligand pairs, however, is their fundamental role in host-defense, regeneration, and other physiological processes, such as haemostasis. For instance, mice genetically deficient for CD40L or wild type mice treated with a blocking anti-CD40L antibody are protected from atherosclerosis (28). However, clinical treatment with a neutralizing anti-CD40L antibody in patients with Systemic Lupus Erythematosus has failed due to a higher rate of thrombotic complications (29). Likewise, inhibition of TNF-α reduces experimental atherosclerosis (30), but long term inhibition of TNF-α in patients increases the risk for opportunistic infections and non-melanoma skin cancers (31). It has therefore been proposed that targeting inflammatory signaling cascades downstream of several pro-inflammatory receptors may overcome some of these limitations.

## TRAFs are Intracellular Adapter Proteins that Regulate Inflammatory Signaling

TNF receptor associated factors (TRAFs) are a family of cytoplasmic molecules that transduce, regulate, and bundle receptor-mediated signaling by distinct classes of cell-surface receptors that can activate or inhibit downstream signaling pathways mostly by canonical and non-canonical Nuclear Factor kappa B (NF-κB) signaling (32). TRAFs associate with TNF receptors (TNFRs), interleukin 1 receptor (IL-1R), Toll-like receptors (TLRs), RIG-I-like receptors (RLRs), NOD-like receptors (NLRs), and receptors for IL-2, IL-17, IFN, and TGF-β (33–37) (Table 1). The biological role of TRAFs is to regulate cell survival, immunity, and inflammation. To this date, seven different TRAFs, TRAF1 to 7, have been described (32, 38). Because of their ability to modulate downstream signaling of the above mentioned pro-inflammatory receptors, and their broad expression patterns among immune and stromal cell types, TRAFs have gained increasing attention as central inflammatory hubs that may become accessible to a therapeutic modulation of inflammatory disease (39, 40).

**Table 1:** TRAFs-associated receptor and ligand pairs.

**TRAF1**		**TRAF2**		**TRAF3**		**TRAF5**		**TRAF6**	
**Receptor**	**Ligand**	**Receptor**	**Ligand**	**Receptor**	**Ligand**	**Receptor**	**Ligand**	**Receptor**	**Ligand**
TNFR1	TNF, LTα	TNFR1	TNF, LTα	TNFR2	TNF, LTα	TNFR1	TNF, LTα	TNFR2	TNF, LTα
TNFR2	TNF, LTα	TNFR2	TNF, LTα	4-1BB	4-1BB ligand	TNFR2	TNF, LTα	CD40	CD40 ligand
4-1BB	4-1BB ligand	4-1BB	4-1BB ligand	AITR	AITR ligand	CD27	CD27 ligand	p75	NGF, BDNF, neurotrophins
AITR	AITR ligand	AITR	AITR ligand	BCMA	APRIL, THANK	CD40	CD40 ligand	RANK	RANKL
BCMA	APRIL, THANK	BCMA	APRIL, THANK	CD27	CD27 ligand	HVEM	LIGHT, LTα	TACI	APRIL, THANK
HVEM	LIGHT, LTα	CD27	CD27 ligand	CD40	CD40 ligand	LTβR	LTβ	TLR2	PAMPs
OX40	OX40 ligand	GITR	GITR ligand	HVEM	LIGHT, LTα	OX40	OX40 ligand	TLR3	dsRNA
p75	NGF, BDNF, neurotrophins	CD40	CD40 ligand	OX40	OX40 ligand	p75	NGF, BDNF, neurotrophins	TLR4	LPS
RANK	RANKL	HVEM	LIGHT, LTα	LTβR	LTβ	RANK	RANKL	TLR7	ssRNA
CD40	CD40 ligand	OX40	OX40 ligand	p75	NGF, BDNF, neurotrophins	TACI	APRIL, THANK	TLR9	CpG DNA
XEDAR	EDA	p75	NGF, BDNF, neurotrophins	RANK	RANKL	NOD1	PAMPs/DAMPs	NOD1	PAMPs/DAMPs
TLR3	dsRNA	RANK	RANKL	TLR3	dsRNA	NOD2	PAMPs/DAMPs, viral ssRNA	NOD2	PAMPs/DAMPs, viral ssRNA
TLR4	LPS	TACI	APRIL, THANK	TLR4	LPS	RIG-I	PAMPs/DAMPs, viral RNA	RIG-I	PAMPs/DAMPs, viral RNA
		CD30	CD30 ligand	TLR7	ssRNA	IL-17R	IL-17	IFNλR1,	IFNλ
		NOD1	PAMPs/DAMPs	TLR9	CpG DNA	IL-6R	IL-6	TβRI, TβRII	TGF-β
		NOD2	PAMPs/DAMPs, viral ssRNA	NOD1	PAMPs/DAMPs	Troy		IL-1R	IL-1β
		RIG-I	PAMPs/DAMPs, viral RNA	RIG-I	PAMPs/DAMPs, viral RNA			IL-2R	IL-2
		IFNAR1	IFN	IL-6R	IL-6			IL-17R	IL-17
		IL-6R	IL-6	IL-17R	IL-17			Troy	
		IL-17R	IL-17						
		Troy							

*TRAFs-associated receptors are shown in the left column while ligands for TRAFs-associated receptors are shown in the right column*.

**Figure 1:**
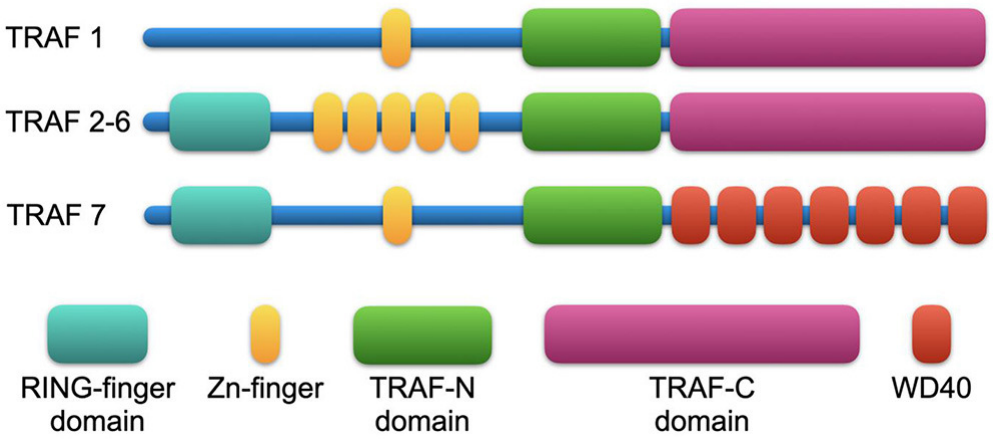
Structural features of TRAF proteins. All TRAF family members (except for TRAF7) share a common domain of 180 amino acids at the C-terminal end, the “TRAF domain,” which consists of two subdomains: a variable coiled-coil TRAF-N domain and a highly conserved TRAF-C domain, known as the Meprin or the TRAF-C homology (MATH) domain, that is required for oligomerization of TRAF proteins and for the interaction with upstream receptors and downstream effector proteins. At the N-terminal end, a Really Interesting New Gene (RING) domain hosts TRAFs E3 ubiquitin ligase function (except for TRAF1). TRAFs also recruit effector proteins by a variable number of Zn-finger domains: TRAF1 and TRAF7 have one Zn-finger domain; TRAF2, 3, 5, and 6 have five Zn-finger domains; and TRAF4 has seven Zn-finger domains. In TRAF7, the TRAF-C domain is replaced by WD40-repeats.

**Figure 2:**
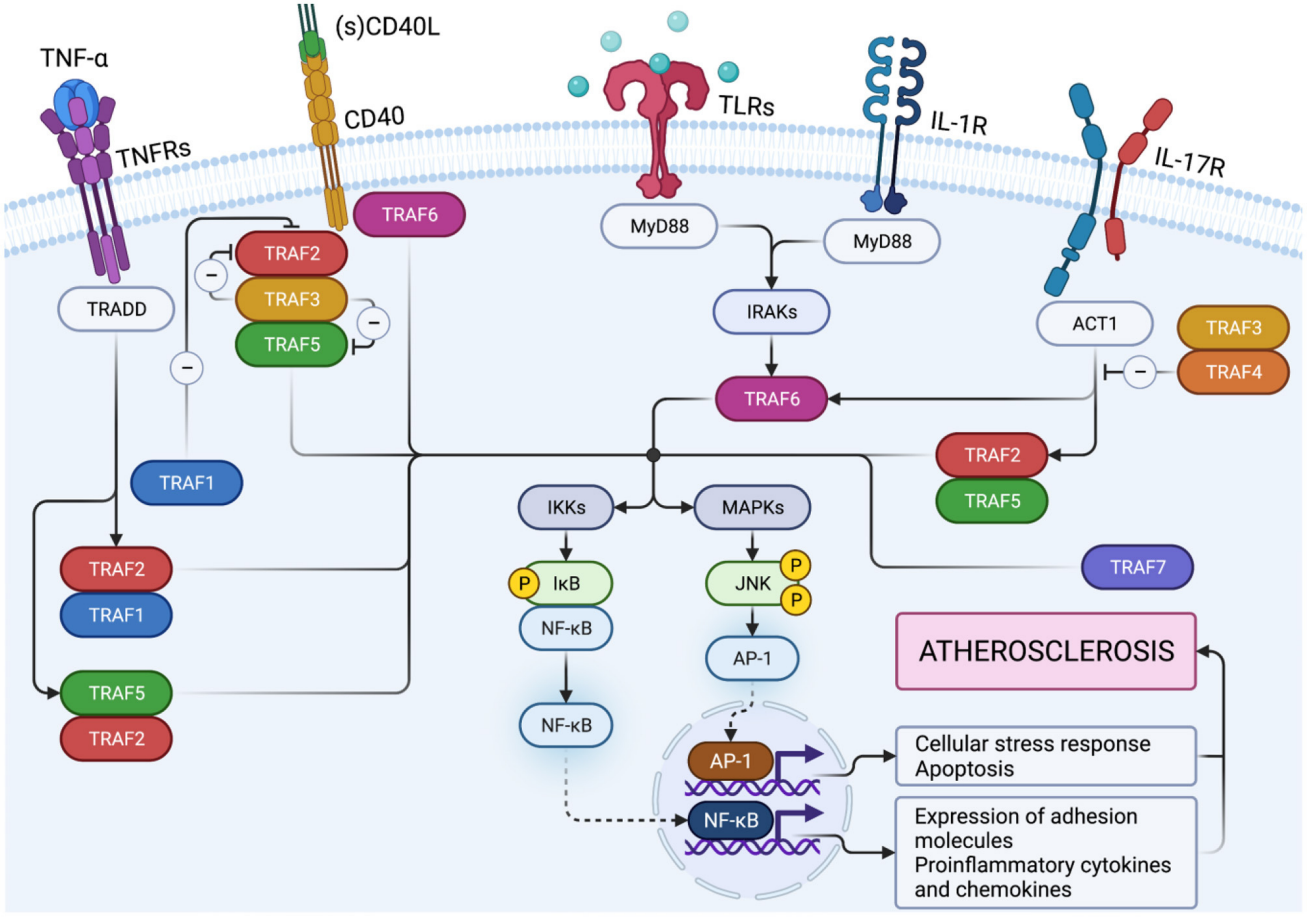
TNF-receptor associated factors (TRAFs) link proximal receptor ligation and distal signaling pathways. Upon TNF-binding, TNFR1 activates inflammatory signaling pathways via TRAF1, TRAF2, and TRAF5. CD40 ligation induces canonical and non-canonical NF-κB activation by TRAF2/5 and canonical NF-κB activation by TRAF6. TRAF3 inhibits NF-κB activation mediated by TRAF2/5 but does not interfere with transcriptional activity of TRAF6-mediated NF-κB. TRAF1 inactivates TRAF2 by direct binding and is considered an inhibitory TRAF. Activation of TLRs and IL-1R can promote MyD88 dependent TRAF6 activity. Following binding of IL-17, heteromeric IL-17RA and IL-17RC recruit TRAF2, TRAF5, and TRAF6 via the adaptor protein Act1 to induce activation of downstream signaling pathways. TRAF3 and TRAF4 on the other hand, inhibit IL-17 signaling by interaction with IL-17R or Act1, respectively. Created with BioRender.com.

Members of the TRAF family are structurally homologous (Figure 1). All TRAFs except for TRAF7 share a common domain of 180 amino acids at the C-terminal end, the “TRAF domain,” which is required for oligomerization of TRAF proteins and for the interaction with upstream receptors and downstream effector proteins (41). The TRAF domain consists of two subdomains: a variable coiled-coil TRAF-N domain and a highly conserved TRAF-C domain formed by seven to eight anti-parallel β-strands, known as the Meprin or the TRAF-C homology (MATH) domain (42). The TRAF-C subdomain allows TRAF molecules to form homo or heterodimers, and to recognize cytoplasmic tails of associated receptors. At the N-terminal end, a flexible α-helical structure mediates downstream signaling and the recruitment of effector enzymes by Zinc- and RING-finger motifs (43). The N-terminal “Really Interesting New Gene” (RING) domain represents the second structurally homologous elements shared by most TRAF members, except for TRAF1. It allows TRAFs to act as an E3 ubiquitin ligase in addition to their scaffolding function (44). Following receptor activation, TRAFs either bind directly to the cytoplasmic tail of the receptor via the TRAF-C domain or indirectly via other adapter proteins such as TNFR type 1 Associated Death Domain (TRADD), Myeloid Differentiation primary response gene 88 (MyD88), or IL-1R Associated Kinase (IRAK). These intermediate signaling adaptors regulate the activation of different kinases including Mitogen Activated Protein Kinase (MAPK) such as JNK, p38, ERK-1/2 (45), and IκB Kinase (IKK). As a result, TRAFs can activate or suppress transcription factor NF-κB and regulate pro-inflammatory cytokine-, chemokine-, and adhesion molecule expression (46, 47). In addition, TRAFs can activate Apoptosis Protein 1 (AP-1), a transcription factor that promotes cellular response to stress (48). Interaction with cellular Inhibitors of Apoptosis Proteins (cIAP1/2) during NOD-like receptor signaling (49) regulates cell survival. Notably, TRAF molecules can interact in homo- or heterooligomers of TRAF family members to increase their avidity and form receptor signaling complexes (40). Importantly, different TRAFs interact with each other to amplify or inhibit distinct inflammatory signaling pathways and may compensate the function of other TRAFs. Given that each TRAF is differentially regulated, interacts with distinct receptors, and activates a variety of downstream signaling pathways, TRAFs represent individual branching points in inflammatory signaling cascades (Figure 2). Different inflammatory stimuli can converge in one TRAF; on the other hand, one inflammatory signal can be divided into different downstream signals through multiple TRAFs (43). This can be exemplified by IL-17R signaling: Upon binding of IL-17, heteromeric IL-17RA and IL-17RC recruit TRAF2, TRAF5, and TRAF6 via the adaptor protein Act1. While TRAF2 and TRAF5 induce activation of MAPKs, TRAF6 mediates activation of NF-κB1, IkBς, C/EBPβ, and C/EBPδ. TRAF3 and TRAF4, on the other hand, inhibit IL-17 signaling by interacting with IL-17RA and IL-17RC or Act1, respectively (40).

## Expression of TRAFs in Atherosclerosis-Relevant Cell Types

**Figure 3:**
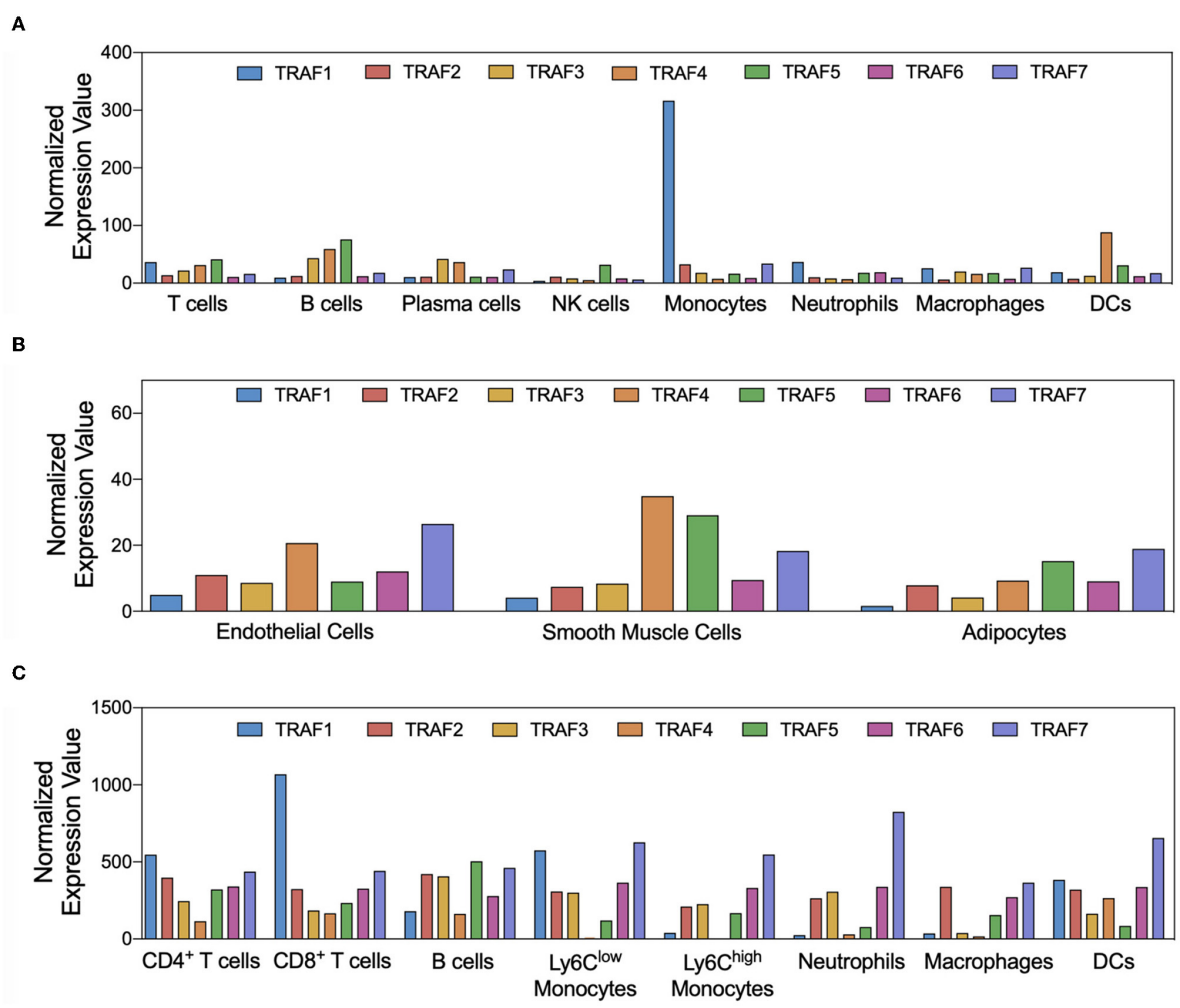
Cell-type specific expression of TRAFs. Gene expression patterns of TRAF1-7 in atherosclerosis-relevant cell types are shown. Normalized expression values for human immune **(A)** and stromal **(B)** cell types were extracted from the Human Protein Atlas project (51), not further adjusted, and plotted as given. Normalized expression of TRAF mRNA in mouse cell types was extracted from the ImmGen database **(C)**.

**Figure 4:**
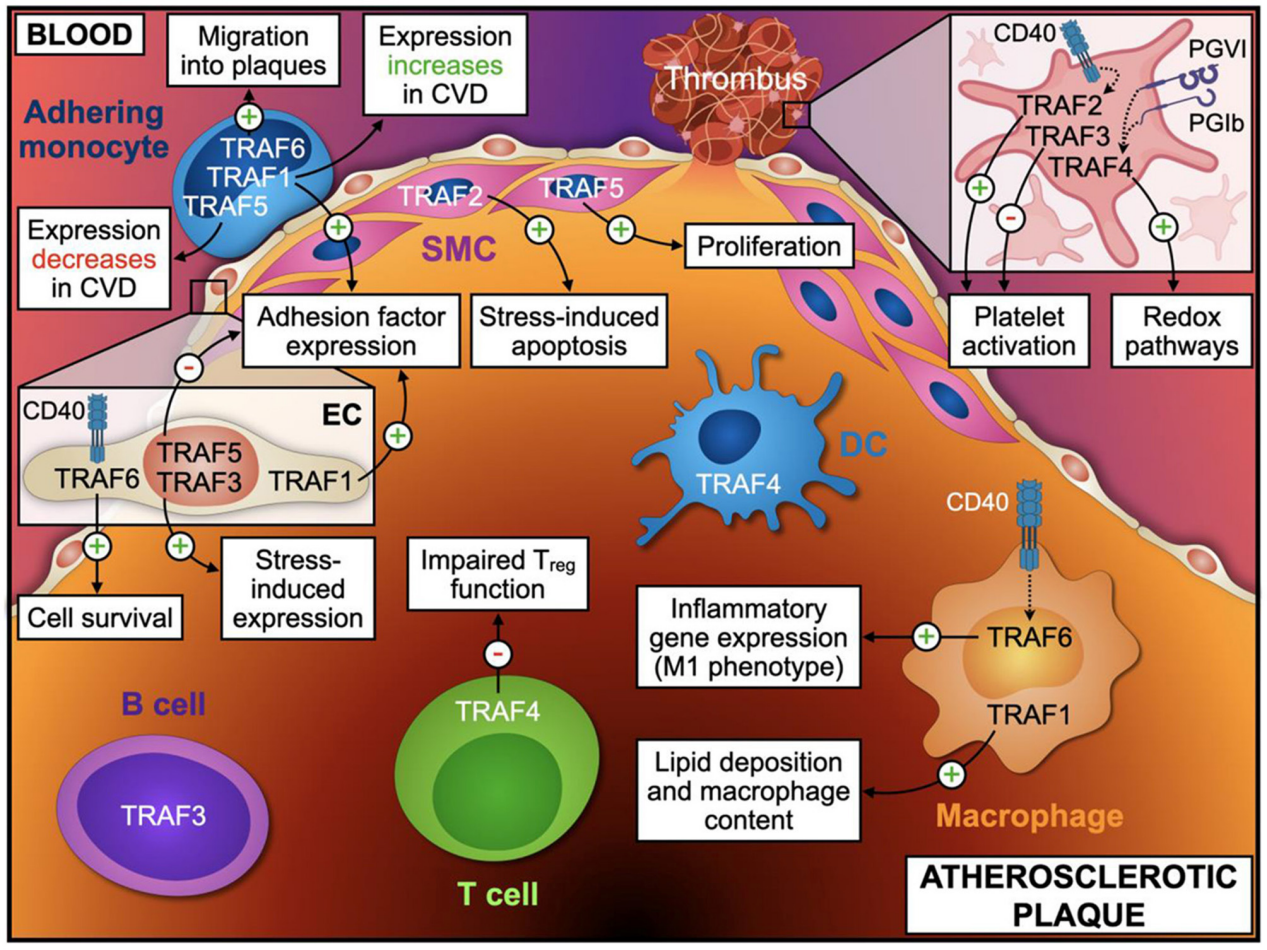
TRAF-effector functions in atherosclerosis. Distinct cellular phenotypes and functions regulated by TRAF-associated signaling modulate vascular inflammation and atherosclerosis. The atherosclerotic plaque is separated from the blood by a thin layer of endothelial cells (EC) and smooth muscle cells (SMC). Monocytes adhere to the vascular endothelium and migrate into the plaque, where they differentiate into macrophages. Additionally, T cells, B cells, and Dendritic cells (DC) accumulate within the vessel wall and take part in regulation of local inflammatory processes. Upon rupture of the fibrotic cap, platelet activation precipitates thrombus formation and subsequent clinical complications. Only TRAFs with a proven effect or validated expression in the respective cell types are shown. Relevant TRAF-dependent processes in EC and platelets are indicated in the inlays. This figure was generated with schematics from BioRender.com.

Immune, endothelial, and smooth muscle cells are the most abundant cell types that build the microarchitecture of atherosclerotic plaques (50). TRAFs have distinct expression profiles across these cell types and other stromal cell types: In the human transcriptome, expression of TRAF1 and TRAF5 is mostly confined to immune cells with the strongest expression in monocytes and lymphocytes, respectively (Figure 3A). Other TRAFs, such as TRAF2, TRAF6, and TRAF7, are ubiquitously expressed in stromal and immune cells. TRAF3 is mostly expressed in glial cells, neuronal cells, T and B cells; TRAF4 in epithelial and trophoblast cells (51). Expression of mRNAs coding for TRAF4, TRAF5, and TRAF7 can be detected in ECs, SMC, and adipocytes (Figure 3B). There is also a considerable variation among different immune cell types: TRAF1, TRAF4, and TRAF5 are the most expressed TRAFs in human T cells, while B cells express high levels of TRAF3, TRAF4, and TRAF5 but not of TRAF1. In human monocytes, TRAF1 is strongly expressed and shows the highest relative expression of all cell types available in the human protein atlas. Human dendritic cells (DCs) contain most TRAF4 transcripts compared to all tested immune cell types, while NK cells are generally only weak TRAF-expressors. Human expression patterns are distinct to mouse transcriptomic atlases, such as the Immunological Genome Project (ImmGen) (52), and suggest differential functional repertoires in mice and humans (Figure 3C). While these findings inferred from TRAF mRNA expression suggest clear patterns of cell type specificities and function, it is important to note that gene expression may not ultimately predict protein expression, post-translational modification, or function. This is highlighted by numerous reports demonstrating functional protein expression of TRAFs in B cells (53), T cells (54), DCs (55), neutrophils (56), macrophages (57), platelets (58), adipocytes (59), SMC (57), and EC (60), even if mRNAs are expressed at only low levels in resting cells. In addition, expression of TRAFs in macrophages, SMC, and EC (57) increases during inflammation. Whether tissue expression of TRAFs is modulated at atherosclerotic predilection sites, such as arterial branching points, or parts of the vasculature that are protected from atherosclerosis in humans, such as the internal thoracic artery (ITA), remains to be investigated (61). In the following sections, we will discuss expression, signaling networks (Figure 2), and functional roles of TRAFs in vascular inflammation and associated pathologies (Figure 4).

## TRAF1

Expression of TRAF1 (46 kDa) is mainly regulated by NF-κB activation (62). While most resting cells lack TRAF1 protein expression, TRAF1 is strongly upregulated in activated immune cells, mostly in mononuclear cells and lymphocytes, as well as in EC and SMC from atherosclerotic lesions (57, 63, 64). In contrast to other TRAFs, TRAF1 lacks a RING-finger domain and expresses only one Zinc-finger domain (32). Intracellular signaling proteins such as TRAF-Interacting Protein (TRIP), Receptor-Interacting Protein (RIP), caspase 8, members of the cellular inhibitors of apoptosis (cIAP) family, as well as TRAF2, interact with TRAF1 (41, 65–68). TRAF1 and TRAF2 were the first TRAFs to be characterized, notably by their interaction with TNF receptor 2 (TNFR2) (69, 70).

TRAF1 contributes to signaling events by TNF-receptor superfamily members such as TNFR1, TNFR2, or CD40, and inhibits TLR-induced TRIFF signaling (71). Members of the TNFR superfamily stimulate cell survival by the activation of canonical NF-κB signaling and pro-inflammatory MAPK pathways (40). TRAF1 supports canonical NF-κB signaling events from TNFR1, TNFR2, CD40, 4-1BB, and LMP-1 by forming heterotrimers with TRAF2 and possibly through stabilizing TRAF2 by inhibiting its degradation by proteasomes (72–74). The complex of TRAF1 and TRAF2 is part of the E3 ubiquitin protein ligase and positively signals TNF-α/TNFR-2-dependent NF-κB and MAPK8/JNK activation and pro-inflammatory gene expression, which is considered a pro-survival event (75, 76). TRAF1, together with TRAF2 and cIAPs, supresses apoptosis (77), which is also supported by the observation that TRAF1 is instrumental for antigen-specific CD8^+^ T cell responses during HIV and influenza virus infection, partially by promoting T cell survival and memory (62, 78). The absence of TRAF1, thus, impairs NF-κB signaling and favors the accumulation of pro-apoptotic signals in the cell, while transgenic over-expression of TRAF1 supports cell survival (54, 79). In hepatic ischemia/reperfusion injury, TRAF1-deficiency is protective through an inhibition of NF-κB mediated inflammation (80). The proposed pro-inflammatory role of TRAF1 has also been substantiated by reports showing attenuated lung inflammation after lipopolysaccharide (LPS) challenge in TRAF1-deficient mice (81). Taken together, these results argue for a pro-inflammatory and anti-apoptotic function of TRAF1. Other findings, however, have yielded contrasting results that question a merely pro-inflammatory role of TRAF1. First, TRAF1-deficiency unexpectedly induced a hyper-proliferative phenotype in T cells after TNF/TNFR2-dependent stimulation in one report (82), suggesting TRAF1 is a negative regulator of TNFR-signaling. A possible explanation is that TRAF1 can restrain non-canonical NF-κB signaling (83) and T cell proliferation (84). Besides its positive regulation of NF-κB -signaling, TRAF1 may also limit NF-κB activation by several less frequent mechanisms (85, 86). In addition, TRAF1 can dampen TLR/NLR-dependent activation of NF-κB by sequestering the linear ubiquitin assembly complex (LUBAC). This process seems to be independent of TRAF2 and TNFRs (87). Notably, TRAF1-deficient mice are more susceptible to an LPS-induced septic shock (87). A single nucleotide polymorphism (SNP) at the C5-locus of TRAF1 is associated with rheumatoid arthritis in humans (88, 89). Patients with a homozygous TRAF1/C5 rs3761847 GG locus show increased mortality in sepsis and malignancies (90), establishing a clinical association with hyper-inflammation. In addition to its anti-inflammatory properties in TLR signaling, TRAF1 has been reported to disrupt the interaction between TRAF2 and CD40, leading to an attenuation of NF-κB activation (76). *In vivo*, TRAF1-deficient mice showed an increased responsiveness to TNF-induced skin necrosis. In this study, the authors accounted TRAF1 as a negative regulator of TRAF2-depending NF-κB activation (82). Down the same line, intratracheal TNF-α stimulation in TRAF1^−/−^ mice exacerbates TNFR1-dependent liver injury (91). In conclusion, TRAF1 seems to exert differential and partially opposing roles in inflammation and apoptosis, likely caused by cell type and receptor-specificity and the precise signaling context.

### Role of TRAF1 in Vascular Inflammation

TRAF1 is overexpressed in murine atherosclerotic lesions and in neointima formation after arterial injury (57, 92). Atherosclerosis-prone LDLR-deficient mice with a genetic deficiency of TRAF1 develop significantly smaller atherosclerotic lesions after 8 or 18 weeks of high cholesterol diet, suggesting a pro-atherogenic role of TRAF1 (60). This decrease of *de novo* atherosclerosis in the absence of TRAF1 is accompanied by a lower content of macrophages and lipids in TRAF1-deficient plaques, an effect likely caused by reduced VCAM-1 and ICAM-1 expression on EC and reduced β1-integrin expression on macrophages. This observation confirms previous studies that found that TRAF1 is required for the expression of ICAM-1 and chemokines in the lung (81). Bone marrow transplantation studies further indicated a requirement for TRAF1 on bone marrow-derived immune cells and stromal cells in maintaining adhesion factor expression and leukocyte recruitment to inflammatory sites (60). Notably, general immune responses seem not to be affected by a lack of TRAF1 (60), as opposed to previous findings that indicated a hyper-proliferative phenotype in T cells with TRAF1-deficiency (82). In humans, expression of TRAF1 is increased in fibrous, atheromatous, and aneurysmal atherosclerotic lesions of carotid arteries (57). TRAF1 blood mRNA expression is elevated in patients with acute coronary syndrome (60). Furthermore, the rs2416804 allele in the TRAF1 gene was associated with carotid intima-media thickness, a marker for subclinical atherosclerosis that predicts subsequent clinical cardiovascular events (93). Likewise, TRAF1 promoted inflammatory pathways through an activation of the ASK1-mediated JNK/p38 pathway in a model of myocardial ischemia/reperfusion (94). Recent results, however, have questioned a mere pro-inflammatory role of TRAF1 in cardiovascular pathologies: In diet-induced obesity (DIO), TRAF1-deficient mice were protected from diet-induced weight gain and associated metabolic derangements by an increased breakdown of lipids in adipocytes and UCP1-enabled thermogenesis (59). Notably, this effect seems to be caused by hyper-inflammation of adipocytes and adipose tissue, and subsequent induction of catabolic pathways. As atherosclerosis is driven by obesity and hyperlipidemia, improved metabolism in TRAF1 deficiency may contribute to its protective properties in atherosclerotic CVD, even in the light of enhanced inflammation.

## TRAF2

TRAF2, a 56 kDa protein, is ubiquitously expressed (69). TRAF2 is involved in TNFR1-, TNFR2-, Receptor Activator of NF-κB (RANK)-, OX40-, and CD40-signaling, and regulates inflammatory responses mediated by NLRs, RIG-I, and other cytokine receptors such as for IL-6R and IL-17R (44). TRAF2 binds to its receptors through the TRAF-N and TRAF-C subdomains (41). The latter also mediate binding to TRAF1 and to itself to form homodimers. The TRAF2 RING-finger domain acts as an ubiquitin ligase in the canonical pathway of NF-κB activation, which activates IKKβ and releases NF-κB (95, 96). In addition, TRAF2 mediates JNK and p38 signaling (44). These processes trigger inflammatory gene expression and promote cell survival (44). Prolonged stimulation of TNFR2 leads to the degradation of TRAF2, which also negatively regulates non-canonical NF-κB signaling (97). Notably, TRAF1 stabilizes TRAF2 and prevents its degradation (62). TRAF2 also contributes to NF-κB activation after CD40 and OX40 ligation, two pro-atherogenic receptors (98–100). Although a deficiency of TRAF2 abolishes TNF-α signaling (101), TRAF2-deficiency results in chronic inflammation characterized by infiltration of activated T effector and T memory cells. This is caused by a dysregulation of the NF-κB pathway (101). TRAF2-deficiency is lethal in mice due to hyper-inflammation caused by TNF-α (98).

### Role of TRAF2 in Vascular Inflammation

Several studies have demonstrated a crucial role of balanced TRAF2 levels in regulating T cell homeostasis, with either increased or decreased TRAF2 levels leading to inflammatory disorders (44). TRAF2 expression is elevated in murine atherosclerotic lesions and neointima after arterial injury (57, 92). Human atherosclerotic lesions express higher levels of TRAF2 compared to healthy carotid arteries and stable atherosclerotic lesions (53). Because TRAF2-defcient cells are highly susceptible to TNF-α induced-death (98), TRAF2-deficient mice show an atrophy of the thymus and the spleen and die prematurely within 14 days after birth (101, 102). Only one study has tested the impact of TRAF2 in experimental atherosclerosis with TRAF^+/−^ mice in bone marrow transplantations using TRAF2-deficient hematopoietic cells from fetal livers to generate viable mice with a deficiency of TRAF2 in hematopoietic cells (103). In these mice, atherosclerotic lesion size was similar to respective controls, suggesting that TRAF2 expressed in immune cells does not affect atherosclerosis (103). Furthermore, mice with a defective TRAF2-CD40 binding site did not show alterations in atherogenesis or in neointima formation after arterial injury (104, 105). The increased expression of TRAF2 in mouse and human atherosclerotic lesions could potentially be explained by the finding that porcine vascular SMC become apoptotic under mechanical stress by a TRAF2-dependent mechanism that activates the pro-apoptotic transcription factors JNK and p38 (106). Importantly, TRAF2 could contribute to plaque destabilization though the induction of SMC apoptosis. However, available data suggest that TNF-dependent anti-inflammatory properties of TRAF2 outweigh pro-inflammatory effects of TRAF2 in leukocytes and other cell types. In a murine ischemia/reperfusion model, cardiac-restricted overexpression of TRAF2 resulted in NF-κB activation and protection from ischemia-induced cardiomyocyte death (107). In murine DIO, hepatocyte-specific deletion of TRAF2 did not affect body weight or hepatic inflammation but attenuates the hyperglycaemic response to glucagon and protects against hyperglycaemia and hyperinsulinemia (108).

## TRAF3

TRAF3 is a 65 kD protein expressed in various cell types, including EC, SMC, fibroblasts, and immune cells (44). Among immune cells, TRAF3 expression is the highest in B cells, plasma cells, macrophages, and T cells. At the N-terminal end, one RING-, five Zinc-fingers, and a leucine zipper domain mediate downstream signaling. TRAF3 inhibits CD27, CD30, CD40, OX40, and Latent Infection Membrane Protein (LMP)-1 induced canonical and non-canonical NF-κB activation mediated by TRAF2/5 (109–114), but does not interfere with transcriptional activity of TRAF6-mediated canonical NF-κB (115). TRAF3 induces the anti-inflammatory cytokine IL-10 after activation of IL-1/TLR-receptors (116). Consistently, mice with myeloid specific TRAF3-deficiency show decreased IL-10 and enhanced IL-6 and IL-12 cytokine levels following an *in vivo* challenge by LPS. In this model, TRAF3-deficiency results in spontaneous multi-organ inflammation (117). Collectively, these findings suggest that TRAF3 is a negative regulator of the above-mentioned pathways (118). Furthermore, TRAF3 plays an important role in the homeostasis of immune cells as murine TRAF3-defcient B cells, T cells, and DCs show a prolonged survival and constitutive activation (119). Mutations in the TRAF3 gene are associated with multiple myeloma and Waldenstrom's macroglobulinemia in humans (53, 120–122). TRAF3-deficient mice are characterized by impaired leukocyte development and die prematurely, likely caused by a permanent activation of non-canonical NF-κB signaling (123).

### Role of TRAF3 in Vascular Inflammation

Previous studies pointed out anti-inflammatory characteristics of TRAF3 in vascular biology. Laminar blood flow, an atheroprotective stimulus, induces TRAF3 expression and inhibits CD40-induced endothelial activation and cytokine production (124). In EC from human atherosclerotic plaques, TRAF3 expression is upregulated in areas with high shear stress and downregulated in areas of turbulent blood flow (125). Overexpression of TRAF3 in EC inhibits endothelial expression of proinflammatory cytokines and tissue factor, blocks DNA-binding activity of the transcription factor AP-1, and prevents CD40-induced endothelial activation (125), a known driver of atherogenesis (126). Silencing TRAF3 expression by small interference RNA (siRNA) in EC increases CD40L-induced cytokine production (53). In addition, TRAF3 expression is upregulated in mice after arterial injury, as well as in murine and human atherosclerotic lesions (57, 92). These data suggest an overall anti-inflammatory role of TRAF3 in vascular inflammation. The fact that TRAF3 expression is elevated in human atherosclerotic lesions (53) may be explained by a compensatory regulation. Interestingly, epigenetic regulation of TRAF3 is associated with vascular recurrence in patients with ischemic stroke (127). Due to the limited viability of TRAF3^−/−^ mice, an *in vivo* atherosclerosis study is missing (128). In cardiac ischemia/reperfusion injury, a knock-down of TRAF3 attenuates infarct size and inflammation by inhibition of NF-κB and xanthine oxidase (XO) signaling pathways and restraining JNK activation (129). In mouse models of high fat diet-induced and genetic obesity, hepatocyte-specific TRAF3 deficiency decreases insulin resistance, hepatic steatosis, and expression of pro-inflammatory cytokines in the liver, while development of obesity *per se* is not altered. These data imply that hepatic TRAF3 has anti-inflammatory properties in the setting of metabolic syndrome (130).

## TRAF4

Unlike other members of the TRAF family, TRAF4 is mostly involved in morphogenic and developmental processes (131). A loss of TRAF4 results in upper respiratory tract deformities and is required for myelin homeostasis in the central nervous system (132). TRAF4 is expressed ubiquitously, but mainly found in thymus, spleen, and lymph nodes. Among leukocytes, B cells, T cells, and DCs express highest TRAF4 levels. Furthermore, TRAF4 is highly expressed in human breast cancer (133, 134). Apart from its stimulating role in DCs migration, TRAF4 does not affect immune cell development (135). In immune cells, TRAF4 increases NF-κB signaling after ligation of the T regulatory (T_reg_) cell expressed “Glucorticoid Induced TNF-receptor” (GITR), which results in an impairment of T_reg_ function (136). Both, GITR (137) and T_reg_ cells (5), have established atheroprotective roles. The role of TRAF4 in TNFR signaling remains unclear, despite a weak association to Lymphotoxin beta Receptor (LTβR) and the nerve growth factor p75 (138). In inflammatory diseases, TRAF4 has been described to exert both, adverse and protective functions: After IL-25/IL-25R ligation, TRAF4 facilitates the Act1/25R interaction and degrades the IL-25R inhibitory molecule DAZAP2, which increases airway inflammation (139). In T_H_17-mediated autoimmune encephalomyelitis, TRAF4 restricts IL-17 induced production of GM-CSF, IL-6, CCL2, and CXCL1 (140). There is only spare information about the role of TRAF4 in atherosclerosis due to the limited viability of TRAF4^−/−^ mice (141). Unstable atherosclerotic human plaques that are prone to rupture and to give rise to thrombotic complications show increased expression of TRAF4 (142). Evidence that TRAF4 is directly involved in vascular inflammation and atherosclerosis is missing.

## TRAF5

The 64 kD protein TRAF5 interacts with multiple inflammatory and atherogenic surface receptors including CD27, CD30, CD40, LMP-1, LTβR, RANK, and OX40 (143–145). It is expressed in lymphoid tissues such as spleen and thymus, but also in epidermis, lungs (146), muscle, and adipose tissue (147). The expression of TRAF5 is highest in immune cells. Structurally, the C-terminal TRAF-domain is responsible for the associations to its receptor and other TRAFs. TRAF5 is highly similar to TRAF2 and TRAF3, and can form heterodimers with TRAF3 to facilitate their recruitment to inflammatory receptors such as CD40 (148, 149). TRAF5 has one RING- and five Zinc-finger domains at the N-terminal end for downstream signaling and activation of JNK and NF-κB (150). Overexpression of TRAF5 in fibroblasts enhances LTβR-dependent NF-κB and JNK signaling, indicating a pro-inflammatory role (143). In patients, a SNP within the TRAF5 gene is associated with rheumatoid arthritis (151). TRAF5 supports T cell immunity against *L. monocytogenes* (152) and enhances LMP-1 induced B cell responses (153). While these findings argue for an overall pro-inflammatory role, other reports have established a regulatory and anti-inflammatory function of TRAF5: TRAF5-deficient ECs show an enhanced activation of JNK after stimulation with TNF-α (103). While TRAF2 binding to OX40 induces NF-κB activation, the interaction of TRAF5 with OX40 seems to have an inhibitory and regulatory role. Following ligation of OX40, a known pro-atherogenic receptor (154), with an agonistic anti-OX40 antibody, TRAF5^−/−^ CD4^+^ T cells upregulated T_H_2-cytokines. The anti-inflammatory and regulatory properties of TRAF5 were further validated in a mouse model of asthma, where TRAF5-deficiency exacerbated lung inflammation by enhanced infiltration of eosinophils and an increased T_H_2 response (155). If and how T_H_2 immunity affects atherosclerosis is still under debate (5). In addition, TRAF5 limits autoimmune encephalitis in mice (156) and is protective in murine experimental colitis (157).

### Role of TRAF5 in Vascular Inflammation

TRAF5 is overexpressed in human and murine atherosclerotic plaques, with a higher expression in fibrous atherosclerotic lesions from human carotid arteries compared to vulnerable lesions (57), suggesting a protective role of TRAF5. Consistently, TRAF5-deficient mice present with increased atherosclerosis, an effect likely caused by enhanced VCAM-1-dependent leukocyte migration and enhanced expression of MCP-1 and C-X-C motif ligand 1 (CXCL1), resulting in accelerated leukocyte recruitment into atherosclerotic lesions (103). In addition, the authors of this study found increased CD36-dependent foam cell formation and detected lower numbers of atheroprotective T_reg_ cells (103). While it has been proposed that TRAF5 can be partially compensated by TRAF2 (158), a deficiency of TRAF5 in bone-marrow derived cells did not affect atherosclerosis in LDLR^−/−^ mice (103), rendering effects on lipid deposition and lower numbers of T_reg_ cells in plaques as indirect effects. An overall anti-atherogenic role of TRAF5 is supported by the fact that TRAF5 mRNA expression is decreased in patients with stable coronary heart disease compared to healthy individuals (103). In another study, the lack of the TRAF2, TRAF3, and TRAF5 binding site on CD40 in MHCII^+^ cells did not interfere with neointima formation after arterial injury and atherosclerosis (104, 105). However, in another report, the lack of TRAF5 protected from neointima formation and vascular SMC proliferation (159). In myocardial ischemia/reperfusion injury, TRAF5 protects against inflammation and associated cardiomyocyte damage via AKT signaling (160). Recently, we have shown that a genetic deficiency of TRAF5 in mice aggravates DIO and its metabolic derangements by a proinflammatory response in adipocytes, pointing toward an anti-inflammatory role of TRAF5 in cardiometabolic disease (147). Down the same line, other reports found that CD40-TRAF2/3/5 signaling in MHCII^+^ cells protects against adipose tissue inflammation and metabolic complications associated with obesity (161, 162). Thus, TRAF5 signaling seems to exert an overall anti-inflammatory effect. TRAF5 reduces atherosclerosis and improves cardiometabolic risk factors. Interestingly, TRAF5 is downregulated during aging (163). Restoring or boosting TRAF5 function may therefore represent a promising therapeutic approach in cardiovascular disease.

## TRAF6

TRAF6, a 63 kD protein, is involved in multiple NF-κB-dependent processes and mediates signaling of the TNFR superfamily members, such as of CD40 and RANK, and members of the IL-1/TLR superfamily (146, 164). TRAF6 was first described as a signaling intermediate of IL-1R and CD40 (164). In addition, TRAF6 seems to be important for signal transduction of IL-17 and IL-25 receptors (165, 166). Overexpression of TRAF6 leads to p38- and JNK-activation by an interaction with TRAF2 (167). TRAF6-deficient mice show severe defects in organogenesis, lack secondary lymphoid organs, have thymic atrophy, and are characterized by decreased numbers of T_reg_ cells and exaggerated inflammatory cell accumulation in most organs (40, 168). CD40 receptor exhibits two distinguished cytoplasmic TRAF-binding sites: a proximal binding site for TRAF6 and a distal binding site for TRAF2, TRAF3, and TRAF5 (169). TRAF6 and TRAF2/3/5 cooperate in the differentiation of B cells (170), while CD40-TRAF6 signaling seemed to be relatively more important in inducing inflammation in a model of murine autoimmune encephalitis (171). A lack of CD40-TRAF6 binding abrogated NF-κB, JNK, and p38 activation, and blunted inflammatory cytokine production after CD40 activation in monocytes and macrophages (172).

### Role of TRAF6 in Vascular Inflammation

Among all TRAFs, TRAF6 has the highest relative expression in human arterial tissue (57), a finding consistent in murine atherosclerotic lesions (57). This modulation seems to be site-specific, since mRNA expression of TRAF6 remains unchanged in blood of patients with or without coronary heart disease (173). TRAF6 deficiency of hematopoietic cells does neither change the size of atherosclerotic plaques in mice nor alters plaque composition (173). This sparked the idea that distinct pro- and anti-inflammatory TRAF6-dependent signaling pathways with a net zero effect would exist. Interestingly, a selective lack of the CD40-TRAF6 binding site, as tested with a chimeric CD40 transgene carrying specific mutations within the TRAF6 binding site in MHCII^+^ monocytes, macrophages, dendritic cells, and B cells (171), reduced neointima formation after carotid arterial injury, whereas the TRAF2/3/5 binding did not affect neointima formation in mice (92, 104). The CD40-TRAF6 axis also contributes to atherosclerosis by an activation of mononuclear cells, whereas CD40-TRAF2/3/5 interactions are crucial in CD40-driven immunity in other cell types, including B cells (174). Mice with a defective TRAF6 binding site on CD40 develop smaller atherosclerotic lesions compared to wildtype and CD40-TRAF2/3/5 deficient mice (105). Mice with a defective CD40-TRAF6 binding site also show reduced numbers of Ly6G^high^ monocytes in peripheral blood, a reduced migration of monocytes to the arterial wall, and increased levels of anti-inflammatory M2-macrophages. This overall atheroprotective phenotype coincides with a higher content of collagen and SMC in atherosclerotic plaques, which resembles a more stable plaque phenotype in humans (105, 175). Beyond this MHCII-restricted effect, endothelial TRAF6-deficiency protects from atherosclerosis in female ApoE-deficient mice by inhibiting NF-κB-dependent proinflammatory gene expression and monocyte adhesion to EC (176). Likewise, murine ECs deficient for TRAF6 display a markedly reduced expression of VCAM-1, ICAM-1, E-selection, MCP-1, and MCP-3 levels after stimulation with oxLDL. Contrastingly, myeloid cell-specific TRAF6-deficiency caused exacerbated atherosclerosis with larger plaques containing more necrotic areas in both in male and female ApoE-deficient mice (176). ApoA-I, the core protein of high-density lipoprotein (HDL) cholesterol suppresses CD40 signaling in macrophages, by preventing TRAF6 translocation to lipid rafts through ABCA1-dependent regulation of free cholesterol efflux, which may present a novel mechanism of ApoA-I-mediated suppression of inflammation in macrophages (177). In vascular SMCs, TRAF6 induces SM22β ubiquitination, which maintains survival through increased G6PD activity and NADP^+^ production (178). Thereby, TRAF6 participates in the regulation of glucose homeostasis during vascular repair after injury (178).

In summary, a growing body of evidence demonstrates a significant involvement of TRAF6-signaling in the pathogenesis of atherosclerotic CVD. Partially opposing results may well be explained by the distinct and cell-type specific expression of TRAF6-associated upstream receptors, i.e., mostly CD40-associated TRAF signaling has been demonstrated to exacerbate inflammation. In this context, the specific targeting of CD40-TRAF6 interactions remains highly desirable. It is noteworthy to mention that a selective blocker of the interaction between CD40 and TRAF6 (“TRAF-STOPs”) that does not affect CD40-TRAF2/3/5 interactions and preserves CD40-mediated immunity reduces atherosclerosis, likely by impairing inflammatory leukocyte recruitment (39). In murine obesity, coinhibitory suppression of T cell activation by CD40 protects against obesity and adipose tissue inflammation, while CD40-deficient mice display an aggravated phenotype during DIO (27). While a genetic deficiency of CD40-TRAF2/3/5 signaling aggravates obesity and promotes metabolic dysfunction and hepatic steatosis, mice lacking the CD40-TRAF6 binding site are protected from obesity-associated complications, arguing for opposite roles of CD40-TRAF2/3/5 and CD40-TRAF6 signaling in obesity-associated metabolic dysregulation. Notably, pharmacologic inhibition of the CD40-TRAF6 pathway ameliorates obesity-related metabolic complications (161).

## TRAF7

TRAF7, a 74 kDa protein, was originally described as a positive regulator of the stress-induced transcription factors AP-1 and CHOP (179). In contrast to other TRAFs, TRAF7 lacks the TRAF-domain and contains seven WD40-repeats at the C-terminal end followed by a RING-finger domain, potentially implying that it evolved separately (40). Expression of TRAF7 is found ubiquitously but relatively higher in the heart, spleen, kidney, liver, colon, skeletal muscle, and placenta (179, 180). Clinically, a TRAF7 mutation causes defects in the heart, intellectual disability, and facial defects (181). TRAF7-dependent p38 and JNK activation contributes to cellular apoptosis, while an overexpression of TRAF7 does not induce activation of NF-κB (38). siRNA targeting TRAF7 show inhibitory and agonistic effects on NF-κB activation depending on the cellular model. TLR2-dependent NF-κB activation is promoted by TRAF6 and TRAF7 (182). Because TLR2 is known to promote atherosclerosis (183), it may be speculated that TRAF7 inhibition impairs atherogenesis. However, no human or *in vivo* animal data addressing the role of TRAF7 in inflammatory or vascular disease have been published to date.

## TRAFs in Platelet Function and Homeostasis

Beyond their known role in homeostasis, platelets have been proposed to contribute to acute and chronic inflammatory pathologies, including atherosclerosis and atherothrombosis in the later stages of the disease (184, 185). Key mechanism of platelets that promote inflammation include the adhesion to the injured endothelium, leukocyte activation, and the formation of leukocyte-platelet aggregates (186). It has also been recognized that platelets have an active secretome, allowing either rapid secretion of pre-formed soluble mediators or *de novo* synthesis upon platelet activation. Major platelet-derived factors are pro-inflammatory, such as RANTES, CXCL4, IL-1β, or CD40L (187). TRAF-associated receptors, such as CD40 and TLRs, have been detected on platelets (188, 189). Ligation of platelet CD40 by CD40L induced platelet activation by yet not clarified downstream signaling pathways (190) that may also involve TRAFs. Notably, expression of TRAFs in megakaryocytes—the bone marrow derived precursors of circulating platelets—was demonstrated by different studies (53, 56). Consistently, platelets express several TRAF members, including TRAF1, TRAF2, TRAF3, TRAF4, and TRAF6 (191). TRAF4 has been identified as a novel binding site for the platelet glycoproteins GPVI and GP1β (58). It has been proposed that engagement of both glycoproteins during platelet adhesion and binding of TRAF4 to their cytoplasmic tail provides a functional link to TRAF4-related downstream effector functions, such as activation of NADPH-related redox pathways (192). Whether TRAFs can be expressed at later stages of platelet differentiation is not clarified yet. It has been shown that soluble CD40L potentiates platelet activation and aggregation through a TRAF2/Rac1/p38 MAPK signaling pathway (193), while TRAF3 was demonstrated to play a negative role in regulating platelet activation (194). Mice with a genetic inhibition of CD40-TRAF6 signaling did not show altered platelet deposition and thrombus formation in an *in vitro* flow chamber assay, suggesting no functional role for TRAF6 in platelet function (195). These data unveil that some TRAFs directly affect platelet function and thereby provide direct signaling links between atherosclerosis and atherothrombosis. However, future studies are required to understand the participation of the TRAF family members in regulation of platelets primary functional repertoire.

## Conclusion and Therapeutic Perspective

TRAFs are potent regulators of several pro-inflammatory and pro-atherogenic signaling pathways (Figure 4), including these initiated by TNF-, IL-1-, IL-6-, IL-17-, and TLR-signaling. Its fundamental role in regulating these signaling cascades in either a pro- or anti-atherosclerotic fashion makes the TRAF family an attractive target for the treatment of atherosclerosis and its complications including myocardial infarction and ischemic stroke. Traditional anti-inflammatory therapies aim for the disruption of one inflammatory signaling cascade, either of soluble ligands or receptors by monoclonal antibodies or small molecule inhibitors, which is exemplified by monoclonal antibodies inhibiting IL-1β (11) or IL-6 (12). Moreover, broad anti-inflammatory therapy with low-dose colchicine has substantiated the role of anti-inflammatory treatments (18). However, these therapies are at the potential risk of side-effects, e.g., of an impairments of host-defense, compensatory up-regulation of other inflammatory pathways, and relapsed inflammation after discontinuation (196, 197). TRAF-directed therapies may be designed to specifically promote or inhibit downstream receptor signaling events. Such therapies may overcome some of the limitations of conventional anti-inflammatory therapies, but raise important considerations as well: First, TRAFs show a highly complex interplay with distinct downstream signaling cascades and associated upstream receptors with partially opposing and overlapping functions. TRAFs complex with other TRAFs and thereby inhibit or augment signaling capacities. Second, this complexity seems to be determined by the levels of cellular expression, concomitant signaling events, and the context of the disease. Third, numerous animal experiments highlight that unspecific neutralization of TRAFs, e.g., in genetic knock-outs, may cause lethal complications and drives unconventional, often unforeseeable signaling events. These conclusions argue for a more careful evaluation of TRAFs in the context of inflammatory disease. However, recent studies have shown that TRAFs can potently limit atherosclerotic and vascular disease. Their broad action on different cell types, stromal, and bone-marrow derived cells, may be of advantage in the multi-factorial and multi-cell type pathogenesis of atherosclerosis. At present, most promising targets for an anti-atherosclerotic therapy are TRAF1, TRAF5, and the CD40-TRAF6 axis as suggested by preclinical mouse models: TRAF1 acts as a pro-inflammatory, atherosclerosis-promoting factor, while TRAF5 serves as an atheroprotective factor; the inhibition of the CD40-TRAF6 axis attenuates atherosclerosis and neointima formation by limiting accumulation of inflammatory monocytes at sites of inflammation. These TRAFs have also demonstrated powerful effects on associated cardiometabolic conditions, such as hepatic steatosis, adipose tissue inflammation, and insulin resistance, which may in part explain atheroprotective effects. The development of small molecule inhibitors that specifically target receptor-TRAF interactions (TRAFs-STOP) demonstrates the feasibility of specific pharmacological compounds to modify TRAF function *in vivo*. However, other TRAFs and their functions remain potentially relevant in vascular biology: TRAF2 induces apoptosis of vascular SMC and promotes plaque destabilization, but lesion size is not affected by TRAF2-deficiency on hematopoietic cells. TRAF3 has some atheroprotective properties, but an *in vivo* mouse study is missing due to the limited viability of TRAF3-deficient mice. To date, there is only little evidence that TRAF4 and TRAF7 influence atherosclerosis, which calls for future investigations. Besides the development of novel, likely cell-type specific TRAF-modulators, it will also be necessary to interrogate the role of TRAFs in atherosclerosis-associated cell types and organs, such as in the bone marrow and platelets. The regulation of TRAFs in human cells, tissues, and disease models represents another important gap of knowledge. Addressing these challenges could result in the clinical development of TRAF-based inhibition of inflammation in future.

## Author Contributions

All authors listed have made a substantial, direct, and intellectual contribution to the work and approved it for publication.

## Funding

This study was funded by the Deutsche Forschungsgemeinschaft (DFG, German Research Foundation), SFB1425, project #422681845. This project has received funding from the European Research Council (ERC) under the European Union's Horizon 2020 research and innovation program (grant agreement No 853425). MCG was funded by the IMM-PACT-Programme for Clinician Scientists, Department of Medicine II, Medical Center - University of Freiburg and Faculty of Medicine, University of Freiburg, funded by the DFG - 413517907.

## Conflict of Interest

The authors declare that the research was conducted in the absence of any commercial or financial relationships that could be construed as a potential conflict of interest.

## Publisher's Note

All claims expressed in this article are solely those of the authors and do not necessarily represent those of their affiliated organizations, or those of the publisher, the editors and the reviewers. Any product that may be evaluated in this article, or claim that may be made by its manufacturer, is not guaranteed or endorsed by the publisher.
